# Fcγ Receptor IIa *(FCGR2A)* Polymorphism Is Associated With Severe Respiratory Syncytial Virus Disease in Argentinian Infants

**DOI:** 10.3389/fcimb.2020.607348

**Published:** 2020-12-18

**Authors:** María Pía Holgado, Silvina Raiden, Inés Sananez, Vanesa Seery, Leonardo De Lillo, Lucas L. Maldonado, Laura Kamenetzky, Jorge Geffner, Lourdes Arruvito

**Affiliations:** ^1^ Instituto de Investigaciones Biomédicas en Retrovirus y SIDA (INBIRS), Universidad de Buenos Aires, CONICET, Ciudad de Autónoma Buenos Aires, Argentina; ^2^ Hospital General de Niños “Pedro de Elizalde”, Ciudad Autónoma de Buenos Aires, Argentina; ^3^ IMPaM, CONICET, Facultad de Medicina, Universidad de Buenos Aires, Ciudad Autónoma de Buenos Aires, Argentina

**Keywords:** infants, severity, virus, antibodies, Fcγ receptor

## Abstract

**Background:**

Most patients with respiratory syncytial virus (RSV) infection requiring hospitalization have no risk factors for severe disease. Genetic variation in the receptor for the Fc portion of IgG (FcγR) determines their affinity for IgG subclasses driving innate and adaptive antiviral immunity. We investigated the relationship between FcγRIIa-H131R polymorphism and RSV disease.

**Methods:**

Blood samples were collected from 182 infants ≤24-month-old (50 uninfected, 114 RSV-infected with moderate course and 18 suffering severe disease). FcγRIIa-H131R SNP genotypic frequencies (HH, HR, RR) and anti-RSV IgG1, IgG2 and IgG3 levels were studied.

**Results:**

Genotypic frequencies for FcγRIIa-H131R SNP were comparable between uninfected and RSV-infected infants. In contrast, we found a significant higher frequency of HH genotype in severe RSV-infected children compared to moderate patients. Among severe group, HH infants presented more factors associated to severity than HR or RR patients did. Furthermore, compared to moderate RSV-infected infants, severe patients showed higher levels of anti-RSV IgG1 and IgG3.

**Conclusions:**

We found an association between an FcγRIIa (H131) polymorphism and severe RSV disease, which points towards a critical role for interactions between FcγRs and immune complexes in RSV pathogenesis. This genetic factor could also predict the worse outcome and identify those infants at risk during hospitalization.

## Introduction

Respiratory syncytial virus (RSV) is the leading cause of childhood hospitalization due to bronchiolitis, mostly in developing countries. It causes an estimated 30 million infections, 3 million hospitalizations and over 100,000 deaths worldwide in children under five annually (Geoghegan et al., 2017). RSV is also a significant cause of respiratory illnesses in immunocompromised and older adults (Falsey and Walsh, 2005; Coultas et al., 2019).

Most children suffer a mild illness while some of them develop severe bronchiolitis but the reasons underlying this remain unclear. Why severity is higher at ages when infants have neutralizing levels of maternal antibodies or why formalin-inactivated RSV (FI-RSV) induced enhanced disease, are interrogates that have not been elucidated yet (Piedra et al., 1993; Acevedo et al., 2019). The combination of viral factors with the host immune response could contribute to severity of RSV disease (Openshaw et al., 2017; Jares Baglivo and Polack, 2019). In particular, antibodies might play a pivotal role by protecting through neutralizing the virus and enhancing cell effector functions. Alternatively, they can contribute to pathogenesis when failing to protect or favoring infection through mechanism such as ADE (Gimenez et al., 1989; van Erp et al., 2017). The current lack of understanding about their mechanisms of actions in RSV infection hampers the design of safe and effective vaccines against RSV.

The biological effect of immune complexes (IC) often depends on their engagement of receptors for the Fc portion of IgG (FcγRs) on the surface of cells such as monocytes, macrophages, neutrophils, dendritic cells and B cells (Ravetch and Bolland, 2001; Pincetic et al., 2014; Anania et al., 2019). Whether T cell express FcγRs is still controversial, but recent studies strongly suggest that a minor fraction of T cells expresses FcγRII (CD32) (Engelhardt et al., 1995; Holgado et al., 2018). The FcγRs found on these cells include FcγRI (CD64), FcγRIIa and IIb (CD32), and FcγRIIIa (CD16) (Anania et al., 2019). Some variations at genetic level affect the functionality of these receptors having implications during inflammatory process. One of the best known functionally relevant single nucleotide polymorphism (SNP) has been described in the extracellular domain of activating FcγRIIa (rs1801274). The gene encodes either a histidine (H) or arginine (R) at amino acid position 131 (Warmerdam et al., 1991). The single expression of an H allele is sufficient for binding to IgG2 and for interact with IgG3 with higher affinity than RR receptors (Parren et al., 1992; Bruhns et al., 2009; Spector et al., 2013).

*FCGR2* genes are located at 1q23, a locus associated with several autoimmune and inflammatory diseases (Roederer et al., 2015). Interestingly, FcγRIIa SNP has been found to affect susceptibility and/or progression of infectious diseases such as invasive pneumococcal or meningococcal disease, severe malaria, dengue and SARS-CoV (Sanders et al., 1994; Platonov et al., 1998; Yee et al., 2000; Cooke et al., 2003; Yuan et al., 2005; Garcia et al., 2010; Dettogni et al., 2015). There is no data on the role of this SNP in RSV context. Because FcγR polymorphisms affect the binding and clearance of ICs and their presence may promote lung-pathology, we analyzed whether infants with H131 genotype are more susceptible to RSV infection and/or severe disease than infants carrying the other variant.

## Subjects and Methods

### Ethics Statement

Our study was approved by the Ethics Committee at the “Hospital de Pediatría Pedro de Elizalde”, Buenos Aires, Argentina, in accordance with the Declaration of Helsinki. Written informed consent was obtained from all donors or legal guardians.

### Subjects

We recruited 182 previously healthy full-term infants younger ≤ 24-month-old hospitalized at the “Hospital de Pediatría Pedro de Elizalde” (Buenos Aires, Argentina), with no underlying conditions (prematurity, congenital heart and/or pulmonar disease) during the 2017–2019 winter seasons. One hundred and thirty-two children were diagnosed with an episode of RSV bronchiolitis (RSV+) confirmed by indirect immunofluorescence of nasopharyngeal aspirates. Clinical disease severity score (CDSS) based on the modified Tal score was employed to classify patients into mild (0–6), moderate (7–9), or severe (10–12) bronchiolitis at the time of sampling (Mejias et al., 2013; Mella et al., 2013). Based on this, 114 infants were classified as moderate patients while 18 infants suffered a severe disease. Fifty infants admitted to the hospital for scheduled surgery and with no history of hospital admission for any respiratory illnesses composed the uninfected group (RSV-). The surgeries were not related with lung pathology and children had no hereditary disorder, cardiac or respiratory chronic condition or hematologic abnormalities. All children were Argentinian currently living at the southern zone of the Greater Buenos Aires (GBA), which includes Buenos Aires city and surroundings. Characteristics of the infants are shown in **Tables 1** and **2**.

**Table 1:** Demographic and clinical characteristics of the study population.

Variable	Uninfected (n = 50)	RSV-infected (n = 132)	*P*
**Demographics**			
Age, months (mean ± SD)	16.1 ± 13.9	8.3 ± 9.2	**<0.0001^c^**
Male sex, n (%)	26 (52.0)	81 (61.4)	0.3116^d^
**Laboratory and radiographic characteristics**			
WBCs/μl, (mean ± SD)	9220.5 ± 881.5	10588.0 ± 4162.8	**0.0228^c^**
Lymphocytes (%, mean ± SD)	50.2 ± 4.0	37.4 ± 15.2	**<0.0001^c^**
Neutrophils (%, mean ± SD)	60.1 ± 3.0	53.0 ± 16.3	**0.0026^c^**
Lobar consolidation, n (%)	N/A	24 (18.3)	
**Disease severity**			
PICU admission, n (%)	N/A	8 (6.1)	
Mechanical ventilation, n (%)	N/A	3 (2.3)	
Coinfections, n (%)^a^	N/A	2 (1.5)	
CDSS, n (%)^b^			
0 – 6	N/A	0 (0)	
7 – 9	N/A	114 (86.4)	
10 – 12	N/A	18 (13.6)	

Data are presented as mean ± SD or n (%). Abbreviations: WBC, white blood cells; PICU, pediatric intensive care unit; CDSS, clinical disease severity score; N/A, not applicable; RSV, respiratory syncytial virus. Significant statistical differences are highlighted in bold.

^a^Coinfections with Adenovirus.

^b^CDSS was calculated using the modified Tal score.

^c^Mann Whitney test.

^d^Fisher’s exact test.

**Table 2:** Demographic and clinical characteristics of moderate and severe RSV-infected infants.

Variable	Moderate (n = 114)	Severe (n = 18)	*P*
**Demographics**			
Age, months (mean ± SD)	8.8 ± 9.8	5.2 ± 4.1	0.127^a^
Male sex, n (%)	71 (62.3)	10 (55.6)	0.610^b^
**Laboratory and radiographic characteristics**			
WBCs/μl, (mean ± SD)	10509.2 ± 3979.9	11140.6 ± 5398.9	0.894^a^
Lymphocytes (%, mean ± SD)	38.0 ± 15.0	33.4 ± 16.0	0.429^a^
Neutrophils (%, mean ± SD)	52.4 ± 16.4	56.8 ± 16.6	0.455^a^
Lobar consolidation, n (%)	18 (15.8)	6 (33.3)	0.097^b^
**Disease severity**			
PICU admission, n (%)	1 (0.9)	7 (38.9)	**<0.0001** ^b^
Mechanical ventilation, n (%)	0 (0)	3 (16.7)	**0.0022** ^b^
Coinfections, n (%)	0 (0)	2 (11.1)	**0.0177** ^b^
CDSS, n (%)			
7	73 (64.0)		
8	30 (26.3)		
9	11 (9.6)		
10		7 (38.9)	
11		6 (33.3)	
12		5 (27.8)	

Data are presented as mean ± SD or n (%). Significant statistical differences are highlighted in bold.

χ^2^, Chi-square; OR, odds ratio; CI, confidence intervals.

^a^Mann Whitney test

^b^Fisher’s exact test.

### Blood Samples

Approximately 0.2 mL of peripheral blood were collected from infants into vacuum EDTA tubes. After being centrifuged for 15 min at 1,000 rpm, plasma was separated and stored at −80 °C until use for antibody detection. Cells were obtained for DNA extraction.

### DNA Extraction

Genomic DNA was extracted from blood samples using the QIAamp DNA blood mini kit (Qiagen). Samples with a ratio OD_260_/OD_280_ between 1.8–2 were included and stored at −20° C until used.

### Single Nucleotide Polymorphism Genotyping

The genomic DNA fragments that harbor the SNP of *FCGRIIA* were amplified using the following primers (Wu et al., 2014): FcγRIIa-sense 5’-TGCCTATAAGAGAATGCTCACA-3’, FcγRIIa-antisense 5’-TCAAAGTGAAACAACAGCCTGACT-3’. Ten to 100 ng of DNA were added into 25 μL solution containing PCR buffer, 1.5mM Cl_2_Mg, 0.2 mM each dNTP, 10 μM specific primers, and 2 U DNA Taq polymerase (Platinum Taq DNA Polymerase Invitrogen). PCR conditions were as follows: 95 °C for 8 min, followed by 35 cycles of 95 °C for 1 min, 56 °C for 40 s and 72 °C 1 min, then finally 72 °C for 10 min. Amplicons were purified and then sequenced using the Big Dye Terminator sequencing kit v3.1 (Applied Biosystems, USA) on an automated sequencer (Applied Biosystems DNA sequencer 3500). Nucleotide sequences were analyzed using BioEdit Sequence Alignment Editor.

### RSV Stock

Human RSV (subtype A, strain Long) was expanded in HEp-2 cells (ATCC CCL-23) as previously described (Raiden et al., 2017) and subsequently purified on 20% sucrose layer at 4 °C and stored at −80 °C until used.

### ELISA

Indirect ELISAs were developed in house for quantifying serum IgG subclasses (IgG1, IgG2 and IgG3) against RSV proteins. Briefly, plates were coated ON at 4 °C with 1 μg/mL UV-inactivated RSV in carbonate-bicarbonate buffer. After blocking, serum samples were diluted in blocking buffer 1:1,000 (for IgG1) and 1:100 (for IgG2 and IgG3) and incubated for 2 h at RT. After washing, plates were incubated for 1 h at RT with biotinylated anti-human IgG1, IgG2 or IgG3 (1:4000, Southern Biotech) and streptavidin-HRP for 1 h at RT followed by TMB Substrate Reagent (BD Biosciences). The absorbance was measured at 450 nm. Specific dilutions of commercial IVIg (50 mg/mL, Laboratorio de Hemoderivados, UNC) were assayed as calibrators. Samples were relativized to an IVIg dilution of 1:5,000, 1:1,000 and 1:100 when detecting IgG1, IgG2 and IgG3 respectively.

### Statistical Analysis

Statistical analysis was performed using GraphPad Prism 7 software. Data normality was evaluated by Shapiro-Wilk test. Genotype and allele distribution were analyzed using the χ2 test. Proportions were compared by χ2 test or χ2 test for trends, as appropriate. Mann-Whitney test was used for comparing immunoglobulin levels among two populations. P values lower than 0.05 were considered statistically significant. Principal component analysis (PCA) was performed to determine the contribution of certain variables analyzed (RSV infection, gender, age, disease severity, pneumonia, mechanical ventilation requirement, IgG levels and *FCGRIIA* genotype) to the total variance among the specific groups of infants. PCA and multivariate analysis were performed using R (www.r-project.org).

## Results

### Characterization of the Study Population

From April 2017 to December 2019, 132 infants with RSV bronchiolitis (RSV+) and 50 uninfected infants (RSV-) were enrolled. The characteristics are summarized in **Table 1**. Blood samples were obtained from all subjects within 24 ± 12 h of admission. The RSV+ group included 114 moderate and 18 severe RSV-infected infants. There were no significant differences in age and sex between moderate and severe RSV-infected infants. All admitted patients needed O_2_ requirement. Those infants from severe subgroup showed significantly more PICU admission compared with moderate patients (*P <*0.0001). Moreover, all infants requiring mechanical ventilation (MV, 16.7%, n=3; *P* =0.0022) or those that presented co-infections with adenovirus (11.1%, n=2; *P* =0.0177) were from severe group. The characteristics of moderate and severe and RSV-infected infants are summarized in **Table 2**.

### FCGR2A Genotype and Respiratory Syncytial Virus Infection

Firstly, we analyzed the allelic frequencies of the reported SNP for FcγRIIa in both uninfected and RSV-infected infants. The frequency for H variant was 0.48 and 0.45 for uninfected and RSV-infected infants, respectively. The polymorphism was in Hardy–Weinberg equilibrium (HWE) (*P* =0.818).

To explore the relation between FcγRIIa genotype and RSV infection, we examined the genotypic frequencies for FcγRIIa among uninfected and RSV-infected infants. The distribution of genotypes was according to those reported in Argentinian population **(** **Table 3** **)** (Galvan et al., 2011). There were no statistically significant differences in FcγRIIa genotypic frequencies (HH, HR and RR) between RSV-infected and uninfected infants nor between each RSV-infected group with uninfected children, showing the absence of association between the genotype and susceptibility to RSV infection.

**Table 3:** *FCGR2A* polymorphism genotype frequencies in uninfected and RSV-infected infants.

	FcγRIIa-131H/R (rs1801274)				χ^2^	P	OR (95% CI)
	HH n, (%)	RH n, (%)	RR n, (%)			**	
Uninfected (n = 50)	14 (28.0)	20 (40.0)	16 (32.0)				
RSV-infected (n = 132)	24 (18.2)	72 (54.6)	36 (27.3)	*vs. Uninfected*	3476.2	0.1759	1.108 (0.6984–1.757)
Moderate RSV (n = 114)	17 (14.9)	62 (54.4)	35 (30.7)	*vs. Uninfected*	4607.2	0.0999	1.269 (0.7914–2.036)
Severe RSV (n = 18)	7 (38. 9)	10 (55.6)	1 (5.6)	*vs. Uninfected*	4936.2	0.0847	0.4615 (0.2081–1.024)
				*vs. Moderate RSV*	8523.2	**0.0141**	2.758 (1.314–5.791)

χ2, Chi-square; OR, odds ratio; CI, confidence intervals. Significant statistical differences are highlighted in bold.

### FCGR2A Genotype and Severity of Respiratory Syncytial Virus Disease

**Figure 1:**
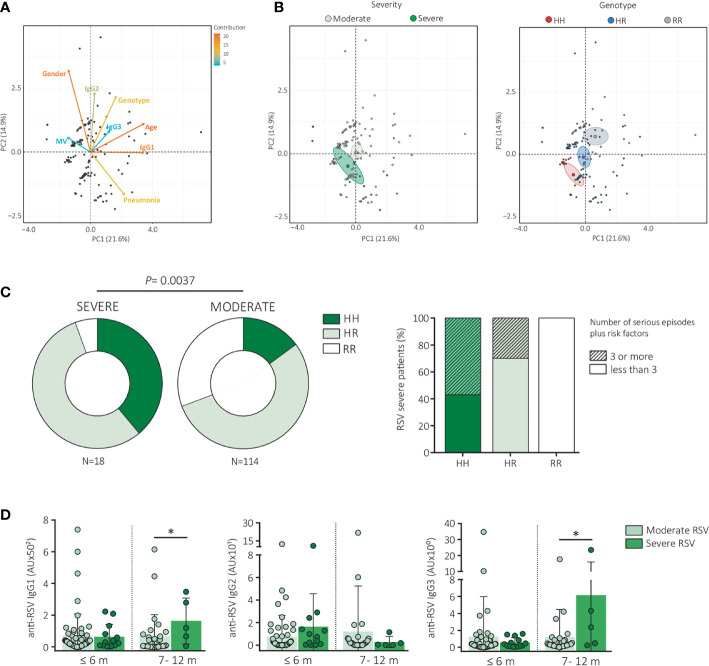
The presence of HH genotype in *FCGR2A* gene is related with the severity of RSV disease. **(A, B)** PCA of nine variables (including genotypic, immunological and clinical parameters) from 132 RSV-infected infants was used to identify the first principal components (PC1 and PC2), which together explain 36.5% of the variation in the data. **(A)** PCA-map showing the sample distribution along the main components (PC1 and PC2) for RSV-infected infants. The color gradient and the size of the vectors represent the contribution of each variable to the sample dispersion in the PCA plot. **(B)** Left panel: PCA-map colored according to disease severity into moderate RSV (light green) and severe RSV (dark green). Right panel: PCA-map colored according to their genotype into HH (red), HR (blue), RR (grey). Individual points represent individual donors. The big circles and ellipses represent the gravity center of each representation code with a confidence interval = 99%. **(C)** Left panel: Donuts graph showing the distribution of *FCGR2A* genotypes among moderate (n=114) and severe (n=18) RSV-infected infants. Chi-square test for trends. Right panel: Stacked bars showing the number of severe episodes and/or risk factors among severe RSV-infected infants divided according to their genotype (HH, HR and RR). Stripped bars: 3 or more serious episodes/risk factors. Smooth bars: less than 3 serious episodes/risk factors. **(D)** Serum levels of IgG1, IgG2 and IgG3 antibodies directed to RSV of moderate RSV (light green) and severe RSV infected infants (dark green) measured by indirect ELISA. Samples were divided according to their age into: under 6-months, between 7 to 12 months. AU., arbitrary units. Mann-Whitney test.

Then, we perform a deeper analysis in order to explore if a genetic compound in combination with immunological and clinical parameters could be related with the outcome of RSV infection and help to distinguish moderate from severe patients. For that, we focused on the 132 infants infected with RSV and performed a multivariate Principal Component Analysis (PCA) based on 9 variables including FcγRIIa genotype, anti-RSV IgG1, IgG2, and IgG3 levels, disease severity, lobar consolidation, mechanical ventilation, age and gender **(** **Figure 1A**). PCA analysis showed that first and second Principal Components (PC1 and PC2) are responsible for the 21.6 and 14.9% of the total variance, respectively. As was expected, gender and age contributed in a high percentage to the clustering among moderate and severe patients. Importantly, FcγRIIa genotype and anti-RSV IgG1 levels also contributed to the difference among RSV subgroups. Furthermore, when we compared the position of the gravity centers grouped by disease severity (**Figure 1B**, left panel) and by FcγRIIa genotype (**Figure 1B**, right panel) it can be seen an overlapped position between the severe patient cluster and HH genotype. In line with this association and because homozygosity for HH is translated into a higher IgG binding capacity than RR, we hypothesized that HH individuals would be at greater risk for severe disease. Indeed, our analysis of chi square (χ2) test for trends revealed that the carriage of a double dose of H allele (HH genotype) was associated with an increased risk of severe RSV disease relative to none carriage (OR = 2.75, 95% CI, 1.31–5.79, *P <*0.0037, **Table 3** and **Figure 1C**, left panel). We found that among severe patients, almost half of the infants were HH (even when we analyzed at each CDSS subgroup: %HH genotype was 42.3, 33.3, and 40.0% for CDSS 10, 11, and 12 respectively), while just a 15% of moderate patients presented this genotype (%HH genotype was 17.8, 10.0 and 9.1% for CDSS 7, 8, and 9 respectively). In addition, we quantified the number of serious episodes/risk factors associated to severity (as lobar consolidation, MV requirement, PICU admission, coinfections with adenovirus, male sex and/or age of 6 months or younger) among severe RSV patients. When we analyzed the distribution of the FcγRIIa genotype, we found that 57% of the infants HH presented a combination of 3 or more of these serious episodes/risk factors. In contrast, only 30% of the HR patients and none RR infants showed 3 or more risk signs (**Figure 1C**, right panel), supporting the idea that carrying the HH genotype is associated with severity characteristics.

As was mentioned before, according to PCA, levels of anti-RSV IgG were some kind contributing to the clustering among moderate and severe patients. To unravel in more detail, the contribution of anti RSV IgG to this segregation, we analyzed the levels of IgG1, IgG2, and IgG3 subclasses. Taking into account the variability that could exist due to different oldness plus the presence of maternal antibodies, RSV-infants were stratified according to their age. Because there were no severe infants older than 12 months to make age-matched comparisons with moderate patients, we grouped RSV-patients into six-month-old or younger and seven to twelve-month-old. Despite the fact that our findings are based on a limited number of severe RSV patients, we found that infants between 7 to 12 months with a severe outcome had higher levels of anti-RSV IgG1 (*P <*0.05) and IgG3 (*P <*0.05) in comparison with moderate RSV children. Even though no significant difference was detected for IgG2 levels, we noticed that severe infants of 6 months or younger presented also higher levels of IgG2 than moderate RSV infants **(** **Figure 1D** **).** These results suggest that anti-RSV IgG might be contributing to severity, although the mechanisms have to be elucidated.

## Discussion

We have explored the relationship between the presence of FcγRIIa H131 polymorphism and the susceptibility to and/or outcome of RSV infection. Our results indicate that the FcγRIIa HH genotype is associated with the risk of develop a severe disease. In a multifactorial pathology as this infection is, this finding may give insight into a genetic factor that could help to predict the severity of RSV.

Locus 1q23, that harbors the *FCGR2A* gene, is well known for its association with a variety of autoimmune and infectious diseases (Roederer et al., 2015). It has been reported that individuals with the RR genotype have a greater risk of developing systemic lupus erythematosus than individuals with the HH genotype (Karassa et al., 2004). Also, this genotype is related with an increased risk of invasive pneumococcal or meningococcal disease, although these findings are controversial (Westendorp et al., 1997; Platonov et al., 1998; Yee et al., 2000; Moens et al., 2006). In the context of the ongoing SARS-CoV-2 pandemic, it is relevant to highlight that RR genotype is also associated with higher severity of SARS-CoV infection (Yuan et al., 2005). On the other hand, presence of HH genotype has been reported to be associated with susceptibility to severe malaria (Cooke et al., 2003) and it was also found enriched in A/H1N1 patients with severe pneumonia (Zuniga et al., 2012). Considering that ICs can have a potential pathogenic role in injuring tissue, the high affinity of HH genotype, instead of contributing to the resolution, may actively cause damage by enhancing the infection and triggering an exacerbated immune response. This deleterious effect of FcγRIIa SNP has been reported for other diseases in which H131 expression is related to progression and/or disease severity (Zuniga et al., 2012).

In this work, we did not find an association between the genotype and susceptibility to RSV infection however, we did observe for the first time an association with the severity. Carrying the FcγRIIa-HH genotype is a heritable risk factor associated with a severe progression once infected, since severe RSV infants showed a significantly higher frequency of HH genotype than moderate RSV infants. Moreover, our results suggest a dose–response relation between genotype and severity, with HH conferring the highest, RR the lowest and HR intermediate association with RSV severity. It could be possible that in a RSV-infection context, a higher affinity of H131 might lead to an increased inflammatory cascade activation after FcγR crosslinking promoting IC-driven pathologies. Interestingly, animal studies support the role of an excessive immune response after RSV infection or the disease enhancement by poorly-neutralizing antibodies observed after immunization with FI-RSV vaccine (Piedra et al., 1993; Polack et al., 2002; Openshaw and Tregoning, 2005). Moreover, it is also known that neutrophils, a cell subset with a high expression of FcγRs are responsible for the initial strong response against RSV that is positively correlated with disease severity (Lukens et al., 2010). In addition, the binding of ICs through FcγRIII and FcγRIIb contribute to RSV pathogenesis by impairing the capacity of dendritic cells to promote IL-2 production thus, affecting the memory CD8+ T cells and regulatory T cell pool (Krishnamoorthy et al., 2012; Raiden et al., 2014; Gomez et al., 2016).

Of note, in spite of FcγRIIa genes are primarily expressed on myeloid and B lymphoid cells, many of the diseases associated to genetic changes in its locus are linked with changes in T cell compartment (Roederer et al., 2015). Indeed, we have recently shown that severe RSV infection in infants is associated with a marked upregulation of CD32 on T cells, whose ligation promotes the activation of CD4+ and CD8+ T cells of hospitalized infants (Sananez et al., 2020).

Severe RSV disease is more prevalent in 6-month-old infants or younger, which mainly rely on maternal IgGs for protection against pathogens. The association between serum IgG levels and protection against RSV disease is poor (Stensballe et al., 2009; Habibi et al., 2015). Primary RSV infections predominantly give rise IgG1 and IgG3 antibodies, whereas subsequent infections only led to an increase in IgG1 and IgG2 titers (Wagner et al., 1989). We found that severe RSV infants between 7- to 12-month-old, have significantly more anti-RSV IgG1 and IgG3 than age-matched moderate RSV infants. Surprisingly, a trend for elevated levels of anti-RSV IgG2 was found in severe infants up to 6-month-old. Whether these antibodies are neutralizing or not, is not clear yet. We have no direct evidence in support of a particular mechanism by which the *FCGR2A* gene polymorphism might influence the severity of RSV infection in this way. However, the fact that IgG1, IgG2 and IgG3 are bound more efficiently by HH-genotype individuals and that this allotype is related with severe disease, suggest that increased amounts of anti-RSV IgG detected in plasma from severe infants could play a potential pathogenic role. Knowing that triggering of FcγRs, including FcγRIIa, on macrophages or dendritic cells may itself lead to a cytokine storm that promotes lung damage during RSV infection is in agreement with this situation (Bohmwald et al., 2019). In addition, has been proposed a potential contribution of IC containing low neutralizing antibodies in ADE of infection during RSV re-infection episodes, asthma and allergies, although it remains to be confirmed.

Finally, regarding the influence of RSV subtype on disease severity, a co-circulation of RSV A and B groups has been reported, being group A the predominant in Argentina. Several studies have found associations between RSV groups, strains, and genotypes with disease severity however the link with severe disease is still controversial (Viegas et al., 2016; Anderson et al., 2019).

Although there is a clear limitation regarding the size of severe population sample, our finding far from being dismissed, should be addressed in future cohorts as it might be of importance in future vaccine and/or prophylactic antibodies development, where would be necessary to shape the T cell response elicited and the subclasses of IgG generated.

In summary, our work shows that the variation in *FCGR2A* gene does not influence the risk to be infected with RSV in infants but it is associated with the outcome of the infection. Importantly, the presence of HH genotype in *FCGR2A* gene related with severe disease may give insight into a genetic factor that could help to identify healthy infants at risk for severe disease at time of hospitalization.

## Data Availability Statement

The raw data supporting the conclusions of this article will be made available by the authors, without undue reservation.

## Ethics Statements

The study was approved by the Ethics Committee at the “Hospital de Pediatría Pedro de Elizalde”, Buenos Aires, Argentina, in accordance with the Declaration of Helsinki. Written nformed consent was obtained from all donors or legal guardians.

## Authors Contribution

All authors contributed to the article and approved the submitted version.

## Funding

This work was supported by grants from the National Agency for Promotion of Science and Technology, Argentina (PMO BID PICT 2016-0444 and PMO BID PICT 2018-2548 to LA), and Roemmers Foundation (to MH and LA).

## Conflict of Interest

The authors declare that the research was conducted in the absence of any commercial or financial relationships that could be construed as a potential conflict of interest.
